# Epigenetics, Stem Cells, and Autophagy: Exploring a Path Involving miRNA

**DOI:** 10.3390/ijms20205091

**Published:** 2019-10-14

**Authors:** Francesca Balzano, Ilaria Campesi, Sara Cruciani, Giuseppe Garroni, Emanuela Bellu, Silvia Dei Giudici, Andrea Angius, Annalisa Oggiano, Vincenzo Rallo, Giampiero Capobianco, Salvatore Dessole, Carlo Ventura, Andrea Montella, Margherita Maioli

**Affiliations:** 1 Department of Biomedical Sciences, University of Sassari, Viale San Pietro 43/B, 07100 Sassari, Italy; mariafrancesca22@virgilio.it (F.B.); icampesi@uniss.it (I.C.); sara.cruciani@outlook.com (S.C.); giugarroni21@gmail.com (G.G.); andrea.angius@irgb.cnr.it (A.A.); vincenzo.rallo@irgb.cnr.it (V.R.); montella@uniss.it (A.M.); 2 Istituto Zooprofilattico Sperimentale della Sardegna, Via Vienna 2, 07100 Sassari, Italy; silvia.deigiudici@izs-sardegna.it (S.D.G.); annalisa.oggiano@izs-sardegna.it (A.O.); 3 Istituto di Ricerca Genetica e Biomedica, Consiglio Nazionale delle Ricerche (CNR), Monserrato, 09042 Cagliari, Italy; 4 Department of Medical, Surgical and experimental Sciences, Gynecologic and Obstetric Clinic, University of Sassari, 07100 Sassari, Italy; capobia@uniss.it (G.C.); dessole@uniss.it (S.D.); 5 National Laboratory of Molecular Biology and Stem Cell Bioengineering of the National Institute of Biostructures and Biosystems (NIBB)-Eldor Lab, at the Innovation Accelerator, CNR, Via Piero Gobetti 101, 40129 Bologna, Italy; carlo.ventura@unibo.it; 6 Operative Unit of Clinical Genetics and Developmental Biology, Viale San Pietro 43/B, 07100 Sassari, Italy; 7 Center for developmental biology and reprogramming-CEDEBIOR, Department of Biomedical Sciences, University of Sassari Viale San Pietro 43/B, 07100 Sassari, Italy

**Keywords:** stem cells, epigenetic, miRNA, autophagy, gender differences, stem cell differentiation

## Abstract

MiRNAs, a small family of non-coding RNA, are now emerging as regulators of stem cell pluripotency, differentiation, and autophagy, thus controlling stem cell behavior. Stem cells are undifferentiated elements capable to acquire specific phenotype under different kind of stimuli, being a main tool for regenerative medicine. Within this context, we have previously shown that stem cells isolated from Wharton jelly multipotent stem cells (WJ-MSCs) exhibit gender differences in the expression of the stemness related gene OCT4 and the epigenetic modulator gene DNA-Methyltransferase (DNMT1). Here, we further analyze this gender difference, evaluating adipogenic and osteogenic differentiation potential, autophagic process, and expression of miR-145, miR-148a, and miR-185 in WJ-MSCs derived from males and females. These miRNAs were selected since they are involved in OCT4 and DNMT1 gene expression, and in stem cell differentiation. Our results indicate a difference in the regulatory circuit involving miR-148a/DNMT1/OCT4 autophagy in male WJ-MSCs as compared to female cells. Moreover, no difference was detected in the expression of the two-differentiation regulating miRNA (miR-145 and miR-185). Taken together, our results highlight a different behavior of WJ-MSCs from males and females, disclosing the chance to better understand cellular processes as autophagy and stemness, usable for future clinical applications.

## 1. Introduction

MiRNA, a small family of non-coding RNA of 19–24 nucleotides are key regulators of orchestrator of different cellular processes, controlling, among others, stem cell behavior [1,2,3,4,5]. MiRNAs act by binding to mRNA sequence sites, causing both mRNA degradation and inhibition of protein translation. In general, a gene can be repressed by more than one miRNA and a miRNA can repress more target genes [2,3]. Human mesenchymal stem (or stromal) cells are multipotent elements, capable of restoring tissue function after injuries, thus representing a source of new cells which are now largely used in regenerative medicine [6,7]. In particular, mesenchymal stem (or stromal) cells from Wharton Jelly (WJ-MSCs) represent a valuable model of these multipotent cells and are easily obtained without ethical issues. In a previous work, we demonstrated that cells isolated from Wharton’s jelly, exhibited gender differences, influencing stemness-related gene expression [8]. In particular, the upregulation of the stemness gene OCT4, was consistent with the upregulation of DNMT1, a crucial epigenetic gene, maintaining DNA methylation during replication [9,10]. Methylation of mammalian genomic DNA by DNMT1, is an important epigenetic modification, which plays central regulatory roles in many cellular processes, including embryonic development, cell reprogramming, and spermatogenesis [11]. OCT4 was previously found to be a transcription factor controlling DNMT1 by binding together with NANOG directly to the DNMT1 promoter, thus inducing its expression [12,13,14,15]. OCT4 induces the expression of DNMT1 responsible for maintaining genes methylated after each cell cycle [15]. Therefore, the expression of genes associated with senescence and developmental regulators are inhibited, keeping cells in a proliferative and undifferentiated state. It is likely that OCT4 can perform the primary function during the first steps of germinal differentiation [12,13,14,15]. Within this context, the development and normal cell performance depend on the interactions with molecules in their microenvironment, including also physical milieu [16,17,18,19]. The main classes of molecules that regulate stem cell development and function include growth and differentiation factors, such as vitamins [20], cell adhesion molecules, and components of the extracellular matrix [21,22], capable of modulating the expression of target genes involving also specific miRNA. In mammals, autophagy is important for pre-implantation development and cell differentiation [21]. In previous studies, it was discovered that HUVEC obtained from male neonates exhibited higher constitutive autophagy, as compared to female cells [22], indicating that the sexual influences on the constitutive autophagic response are cell specific [23]. It is largely known that autophagy, a conserved lysosomal degradation pathway, is highly active during differentiation and development. The path of autophagy is a catabolic process that responds to environmental and hormonal stimuli, guiding the rapid cellular changes needed for a suitable differentiation [24].

Within this context, identifying how OCT4 and DNMT1 genes are mutually and/or contemporary modulated by a common player could reveal more molecular details on specific gender features of WJ-MSCs [8] for future application in regenerative medicine. For this reason, in the present paper, we evaluate the expression of selected identified miRNA, related to stemness and adipogenic and osteogenic differentiation, trying to establish the molecular basis of the previously described gender differences.

## 2. Results

### 2.1. miRNA Expression in WJ-MSCs and Gender Differences

Specific miRNA related to the selected genes (DNMT1 and OCT4), also involved in adipogenic and osteogenic differentiation, were identified in silico and further confirmed by literature [25,26,27,28,29,30,31,32]. In particular miR-185-3p and miR-148a-3p targeted DNMT1 (Table 1), whereas miR-145-5p was identified to target OCT4 gene (Table 1). [25,26,27,28,29,30,31,32,33,34,35,36].

**Figure 1:**
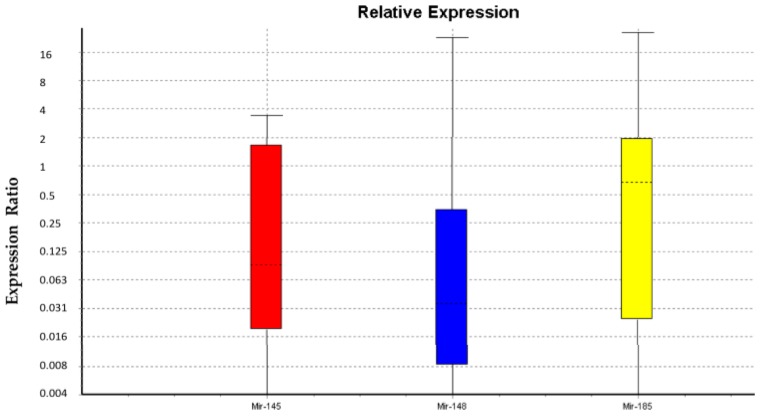
Expression of miRNAs. The graph shows a trend in miRNA expression: miR-145-5p, miR-148a-3p, and miR-185-3p of Wharton jelly multipotent stem cells (WJ-MSCs) from females as compared to WJ-MSCs from males.

Statistical analysis showed that data were not normally distributed. We analyzed three miRNAs using U6snRNA as a normalizer. The Kruskal-Wallis test showed no significant differences between males and females for U6snRNA, miR-145-3p, and miR-185-3p only. Statistical analysis supported our decision to use U6snRNA as a normalizer for PCR analysis in real time due to its greater stability between groups of samples. Figure 1 and Table 2 show the relative expression of miR-145-5p, miR-148a-3p, and miR-185-3p in female WJ-MSCs as compared to males WJ-MSCs. The results show that there are no significant differences in the levels of miR-145-5p and miR-185-3p between males and females. On the other hand, the expression level of miR-148a-3p is significantly lower in males as compared to females.

### 2.2. Adipogenic and Osteogenic Differentiation is Similar in Males and Females WJ-MSCs

**Figure 2:**
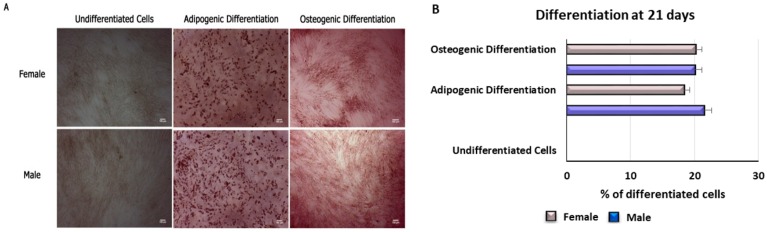
Differentiation of WJ-MSCs after 21 days. (**A**) Differentiation of female and male WJ-MSCs after treatment in specific adipogenic or osteogenic differentiation medium. Undifferentiated cells are WJ-MSCs cultured in basic growing medium. Scale bar = 100 µm. The percentage of differentiation (**B**) was calculated using ImageJ, with WJ-MSCs cultured for 21 days in osteogenic or adipogenic medium. Data are expressed as mean ± SD and are representative of n different experiments. An average was made from three technical replicates.

After 21 days of differentiation, the morphology of WJ-MSCs was evaluated by light microscopy (Leica, Nussloch, Germany). Figure 2 shows that cells cultured in an adipogenic medium exhibited a typical morphology of mature adipocytes (Figure 2). However, we did not observe significant differences between males and females in the time needed for the acquirement of an adipogenic phenotype (Figure 2).

After 21 days of treatment with osteogenic conditioned medium (ODM), calcium accumulation was evident in both males and females. The percentage of mineralization (B) was calculated using ImageJ, with WJ-MSCs cultivated for 21 days in an osteogenic medium as a positive control (black bar), considered as 1, and WJ-MSCs cultivated in basic medium (white bar) as a negative control of differentiation. The images show that no significant differences can be detected between males and females for the osteogenic differentiation.

### 2.3. Autophagy Exhibit Gender Differences in WJ-MSCs

**Figure 3:**
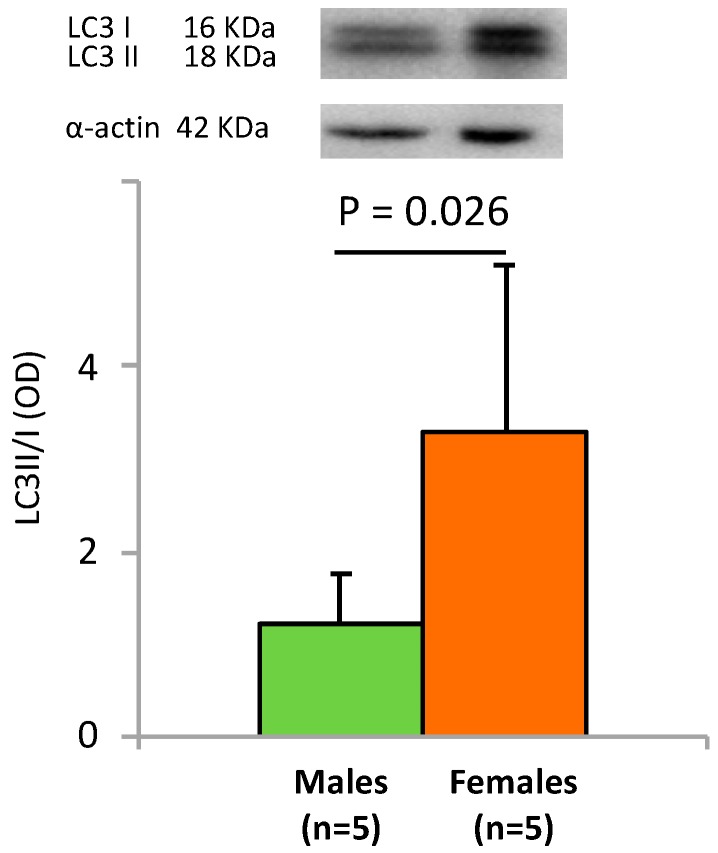
LC3 II/I ratio autophagic marker. Representative Western blot and densitometric analysis of LC3II/LC3I ratio in male and females WJ-MSCs. Values are expressed as means + SD from at least 5 experiments and normalized to actin levels.

The autophagic marker LC3II/I ratio [33,34,35], detected by Western blot analysis, was significantly higher in female cells than in male ones, indicating a higher constitutive autophagy in female cells (Figure 3).

## 3. Discussion

Recent findings indicate that the balance between stem cell self-renewal and differentiation, a crucial event regulating stem cell function [36,37,38,39,40], is finely modulated by epigenetic networks, although specific interplays are still not fully understood [36]. Within this context, in a previous paper, we demonstrated that the expression of the stemness gene OCT4 and the epigenetic modulating gene DNMT1 were significantly higher in WJ-MSCs isolated from males than in those isolated from females [8]. Here, we found that the expression of miR-148a in WJ-MSCS from males is significantly lower as compared to WJ-MSCs from females (Figure 1). Lopez-Bertoni and Ping Wang showed that Oct4 overexpression can downregulate miR-148a by DNA methylation [26,27]. Other authors described that a reduced expression of miR-148a induces higher levels of DNMT1 [25,26]. Further studies revealed that miR-148a directly regulates the expression of DNMT1 by targeting the 3′-UTR of its transcript [25]. Our results, showing that the expression of miR-148a is lower in male WJ-MSCs than in female cells, whereas OCT4 and DNMT1 were overexpressed [8], perfectly fits with this scenario. Moreover miR-148a is involved in autophagy activation, also inhibiting cell proliferation and inducing apoptosis [28]. MiR-148a regulates the autophagy in mammals through the Hedgehog (Hh) signaling pathway that inhibits the synthesis of autophagosomes [34,35]. Constitutive autophagy, measured by the LC3II/I ratio, [28] is significantly different in males as compared to females WJ-MSCS, being higher in the last ones. Interestingly, the LC3II/I ratio change depends on the type of cells isolated from the umbilical cord. In fact, in HUVECs, the ratio is significantly higher in males, whereas no differences are detected in VSMC [34,35,36,37,38].

Among the miRNA described for their effects on stem cell differentiation miR-145 has been defined in particular as a negative regulator of adipogenesis, while targeting KLF4 stemness gene [41].

In the experiments described here we did not find any significant difference between males and females in the expression of miR-145, thus confirming that adipogenic differentiation occurs in the same fashion in males as in females (Figure 2).

It has been previously described that miR-185 negatively regulates in vitro and in vivo osteogenesis as a direct target of miR-185, as miR-185-knockout (KO) mice exhibited an improved osteogenesis through the BMP/Smad pathway activation [31].

Here, we highlight the same trend of expression of miR-185 and miR-145 in WJ-MSCs isolated from males and females, further supporting the undetectable gender differences in the yield of osteogenic and adipogenic differentiation.

## 4. Materials and Methods

The study included umbilical cords (*n* = 12; 6 males and 6 females) retrieved from healthy full-term women. Donors aged between 25 and 35 years, the recruitment criteria were spontaneous birth, donors free from drugs, smoking and diseases.

### 4.1. WJ-MSCs Isolation and Culture

Fresh human umbilical cords (*n* = 12) from both sexes were collected after birth by the Natural Childbirth Section in the Gynecologic and Obstetric Clinic, University of Sassari. The patients gave written informed consent according to the approval of this study by the Ethics Committee (Ethical Clearance No.: 0021565/2018, 22 May 2018—Commissione Etica CNR). The umbilical cords were collected in phosphate buffer saline (PBS) supplemented with 200 U/mL penicillin (Euroclone, Milan, Italy), 200 mg/mL streptomycin (Euroclone, Milan, Italy) and 4 mg/mL amphotericin B (Gibco Life Technologies, Carlsbad, California, USA) prior to storage at 4 °C for further WJ-MSCs isolation. Tissues were dissected into small pieces and then washed with an equal volume of PBS (200 U/mL penicillin, 200 mg/mL streptomycin and 4 mg/mL amphotericin B). The suspension was centrifuged at 300× *g*, 4 °C for 10 min and supernatant was discarded. The precipitate (mesenchymal tissue) was digested with collagenase type I (2 mg/mL) Sigma at 37 °C for 16–18 h with agitation. After neutralization of the enzyme with 10% fetal bovine serum (FBS) (Life Technologies, Grand Island, NY, USA) and filtering (70 μm cell strainer) (Euroclone, Milano, Italy), samples were centrifuged at 600× *g* for 10 min to separate distinct cell fractions. The MSCs from WJ-MSCs were immunomagnetically sorted for c/kit using a monoclonal anti-c/kit (CD117) antibody (Miltenyi Biotech, Minneapolis, MN, USA) directly conjugated to microBeads (Miltenyi Biotech, Bergisch Gladbach, Germania) and then expanded in subconfluent conditions in a basic medium (BM), Dulbecco’s Modified Eagle’s Medium (DMEM) (Life Technologies Grand Island, NY, USA) supplemented with 10% fetal bovine serum (FBS) (Life Technologies, Grand Island, NY, USA), 200 mM *L*-glutamine (Euroclone, Milan, Italy), and 200 U/mL penicillin—0.1 mg/mL streptomycin (Euroclone, Milano, Italy), and plated in 12 cm^2^ flasks filled with this medium. The flasks were placed in the culture incubator at 37 °C with 5% CO_2_ and saturated humidity for 10–14 days [8]. After 48 h of incubation, the cultures were washed with PBS and kept in the fresh medium. The culture medium was changed every 3 days. When the cells reached 80%–90% confluence, they were harvested using 0.25% Trypsin EDTA (Euroclone, Milano, Italy), counted, and transferred into new flasks. The WJ-MSCs used in this study were stained positive for CD90, CD105, CD44, and CD29, typical of mesenchymal stem cells, and negative for CD34, CD133, and CD45. Cells were prepared for the flow-cytometry analysis according to previously described method [8] using a flow cytometer (FACSCalibur, Becton Dickinson, San Jose, CA, USA), by collecting 10,000 events and the data analyzed using the Cell Quest Software (Becton Dickinson); data not shown.

#### 4.1.1. RNA Extraction and Quantitative Polymerase Chain Reaction

RNA was extracted from cells using Mirvana MIRNA ISO Kit 10-40ISO (Life Technologies, Carlsbad, California, USA) according to the manufacturer’s instructions, with the final elution volume of 15 μL. Three individual miRNAs (hsa-miR-148a-3p, hsa-miR-183-3p, and hsa-miR-145-5p) were selected based on amplification efficiency and previous studies by other authors [25,26,27,28,29,30,31,32].

#### 4.1.2. List of Software for the Identification of miRNAs

We employed highly predictive software to identify miRNAs that may influence the expression of the OCT4 and DNMT1 genes. To achieve the best prediction, it is advisable to use the consensus of several software and databases that uses different parameter for target prediction (miRTarBase, miRanda, DIANA-TarBase v8, PITA, TargetScan 7.2, miRDB, miRBase 22) [42] to limit the number of false positive results. The list of software and miRNA associated with the target genes are shown in Table 1 and used for molecular screening. To verify the relationship between miR-185-3p and miR-148a-3p and DNMT1, we searched in PubMed and Scopus databases [25,26,27,28,29,30,31,32]. Based on these findings, DNMT1 is a miR-185 and miR-148a target gene. MiR-145-5p was identified to target OCT4 gene by the miRTARbase database and TARBASE v.8 software [43,44].

#### 4.1.3. Quantitative PCR Analysis

The concentration level of mature miRNAs was tested by quantitative real-time PCR (qPCR), using TaqMan^®^ MicroRNA Reverse Transcription Kit (Life Technologies, Carlsbad, California, USA), for the reverse transcription. TaqMan^®^ Universal Master Mix II, Life Technologies, was used for the PCR according to the manufacturer’s instructions; 45 amplification cycles were performed. miRNA concentration levels were quantified using the IQ5, BIORAD, instrument (Milan, Italy). The U6snRNA was used for the data normalization [45,46]. Real-time PCR was done in duplicate. The sequences and the identification symbols were retrieved from miRbase and are reported in Table 3 [47].

#### 4.1.4. Statistical Analysis and Real-Time PCR Data Analysis

The raw Ct values for each miRNA and U6snRNA were checked for normal distribution. The Kruskal-Wallis test was applied to compare the groups in each target. All the analyses and graphics were performed with SPSS software, version 17.0. Reverse transcription followed by polymerase chain reaction (RT-PCR) is the most suitable method for the detection and quantification of miRNA. It provides high sensitivity, good reproducibility, and wide-range quantification. Several mathematical algorithms have been developed to calculate a ratio of expression based on real-time PCR efficiency and the crossing point deviation of an unknown sample against a control. Then, a software tool named REST© (relative expression software) [48] was used to compare two groups, with a maximum of 16 data points in a sample and 16 in a control group for the reference and up to four target genes. The mathematical model used is based on the PCR efficiencies and the crossing point average gap between sample and control. Subsequently, the reported expression or concentration of transcripts investigated is tested for significance by a randomization test. The relative concentration of the mature miRNAs was analyzed using the software REST. The nonparametric bootstrapping test was used to evaluate concentration differences of miRNAs between samples male and female.

Data were analyzed using Statistical Package for the Social Sciences version 13 Software (SPSS Inc., Chicago, IL, USA). Krustal-Wallis rank-sum and Wilcoxon signed-rank test were applied to evaluate the distributions of each group variance at different times of observation, assuming *p*-value < 0.05 as statistically significant.

Male and female results were compared by statistical analysis. Intra-sexual analyzes were also conducted to compare the results between control cells and cells under study. Continuous parametric variables were analyzed using Student’s unpaired *t*-test. Nonparametric continuous variables were compared with the case of normally distributed samples and with the median ± median absolute deviation (MAD) in the case of non-normal distribution of the samples. The correlations were tested with the Pearson test for normally distributed samples. For all tests, a *p*-value ≤ 0.05 is considered statistically significant.

### 4.2. Analysis of Cellular Mechanisms in WJ-MSCs from Males and Females

#### 4.2.1. Osteogenic Differentiation: Alizarin Red Assay

Cells were cultured for 21 days on tissue culture plate 24 wells (BD-falcon), in the presence of one of the following differentiation media: osteogenic (ODM), adipogenic (ADM), or only basic medium. Positive control (CTRL+) was represented by WJ-MSCs cultured in osteogenic medium. Samples were fixed with 10% formalin for 15 min at RT, washed three times in distilled water (ddH_2_O), and then were stained with 2% alizarin red S solution (Santa Cruz Biotechnology, Dallas, Texas, USA) for 20min at RT. Cells were thoroughly washed several times in ddH_2_O to avoid excess of solution and observed by light microscopy to analyze calcium deposition. The analysis of mineralization was performed using image analysis software (ImageJ, National Institutes of Health, USA).

#### 4.2.2. Adipogenic Differentiation: Red Oil Assay

Cells were cultured for 21 days on tissue cultured in 24 wells (BD-falcon) in Differentiation Medium. After 21 days, cells were fixed for 30 min at RT in 10% formalin, then washed twice in H_2_O and in 60% isopropanol for 5 min. Cells were stained for 15 min in oil red solution, washed once with H_2_O and counterstained for 2 min in Mayer’s hematoxylin solution. Cells were then washed and adipogenesis was evaluated by light microscopy. The analysis of lipid accumulation was performed using ImageJ, using adipocyte cells as a positive control.

### 4.3. Autophagy Detection: Western Blotting

Cells were cultured until confluence and then lysed using cell lysis buffer (Cell Signaling Technology, Danvers, MA, USA), following manufacturer instructions. The protein concentration was quantified using the BCA protein assay kit (Thermo Scientific, Waltham, MA, USA). For the Western blot analysis, 30 μg of solubilized proteins were electrophoretically resolved by 4%–15% SDS-PAGE (120 V, 2 h, room temperature) and then transferred to a PVDF membrane (130 mA, 7 min,) using a mini-PROTEAN tetracell system (Bio-Rad, Milano, Italy). The membranes were blocked in 5% (*w*/*v*) skim milk (Sigma-Aldrich, Milano, Italy) in TBS-tween buffer at RT for 1 h. Membranes were then probed overnight at 4 °C with microtubule-associated protein 1 light chain 3 antibody (LC3-I and LC3-II) (MBL, Milano, Italy) (1:500). After washing, the blots were incubated for 1 h with horseradish peroxidase (HRP)-conjugated secondary antibody (Cell Signaling Technology, Milano, Italy) (1:2,000). Antibody binding was detected using an enhanced chemiluminescence detection system (Cell Signaling Technology, Milano, Italy). Antibody binding was detected using a chemiluminescence reaction (Cell Signaling Technology, Danvers, MA, USA) with the Bio-Rad Chemi Doc instrument (Berkeley, CA, USA). Band volume analysis was performed using the Image Lab 4.0 software (Bio-Rad Laboratories, Berkeley, CA, USA).

Data were reported as the mean ± SD. Statistical analysis was performed by unpaired Student’s *t*-tests to compare cells from males and females. The distribution of samples was assessed by the Kolmogorov-Smirnov and Shapiro tests; *p* ≤ 0.05 was considered statistically significant.

## 5. Conclusions

Epigenetic modulation of stem cell fate is already known, although the specific role of selected miRNA needs further investigation.

In the present paper we started from our previous results concerning gender differences in the pluripotency and epigenetic regulating genes, OCT4 and DNMT1, respectively, in the attempt to better define the different behavior of males and female WJ-MSCs under differentiating conditions.

**Figure 4:**
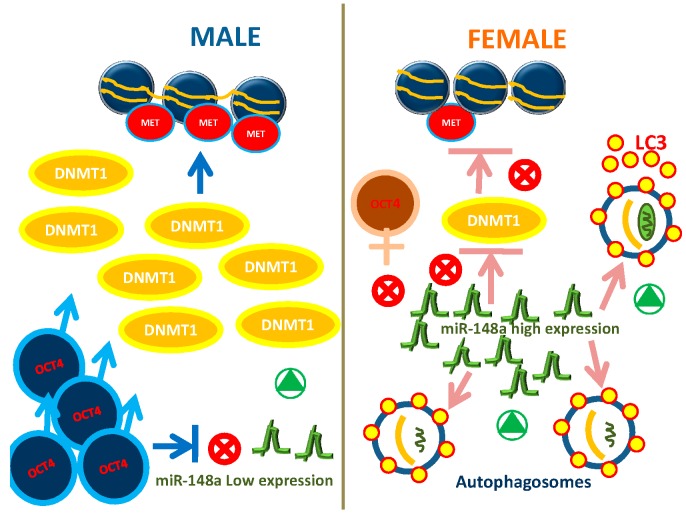
Gene expression networking miR-148a/DNMT1/OCT4/autophagy in WJ-MSCs from males compared with WJ-MSCs from females.

For this reason, we selected specific miRNA playing crucial roles in both stemness regulation and osteogenic and adipogenic differentiation. Considering our results, we speculate the existence of a regulatory circuit involving miR-148a/DNMT1/OCT4/autophagy in WJ-MSCs which could be differently modulated according to gender (Figure 4). It could be interesting to evaluate these variations in stem cells isolated from different tissues during adulthood and to clarify if stem cell differentiation toward other specific phenotypes could be gender influenced. Unraveling this issue could prompt the development of novel strategies in regenerative medicine, giving answer and accelerating translational application for autophagy-related disorders, as neurodegenerative disease [49]. Nevertheless, the different autophagic activities between males and female WJ-MSCs, discovered by us suggest gender differences in embryonic development, offering a valuable on-going in vitro model to study early stages of development.

## Figures and Tables

**Table 1:** Target genes and miRNAs selected using the indicated bioinformatic tools.

Gene miRNA Software	Databases
DNMT1	hsa-miR-148a-3p miRTarBase, Targetscan, DIANA-TarBase, miRBD
DNMT1	hsa-miR-185 miRanda, Targetscan, PITA
OCT4	hsa-miR-145-5p miRTarBase, DIANA-TarBase

**Table 2:** Relative expression of miRNAs (WJ-MSCS by females compared to WJ-MSCs of males): standard error, 95% confidence interval (CI), and *p*-value of miRNAs.

Relative Expression Results							
**Parameter**	**Value**						
Iterations	2000						
**Gene**	**Type**	**Reaction Efficiency**	**Expression**	**Std. Error**	**95% C.I.**	**P(H1)**	**Result**
miR-145	TRG	1.0	0.099	0.009-2.083	0.001-3.079	0.064	
miR-148	TRG	1.0	0.059	0.005-1.508	0.001-22.627	0.039	**Down**
miR-185	TRG	1.0	0.326	0.006-7.205	0.001-25.813	0.419	
US6	REF	1.0	1.000				

MiR-145 sample group is not different to control group, P(H1) = 0.064; MiR-148 is down regulated in sample group (in comparison to control group) by a mean factor of 0.059 (S.E. range is 0.005-1.508), MiR-148 sample group is different to control group, P(H1) = 0.039; MiR-185 sample group is not different to control group, P(H1) = 0.419.

**Table 3:** miRNA collection: accession number, symbol, sequence, and identification number used in this study of miRNA analyzed.

Accession ID Number Symbol Sequence
MIMAT0000243 hsa-miR-148a-3p UCAGUGCACUACAGAACUUUGU
MIMAT0004611 hsa-miR-185-3p AGGGGCUGGCUUUCCUCUGGUC
MIMAT0000437 hsa-miR-145-5p GUCCAGUUUUCCCAGGAAUCCCU

## Abbreviations

MDPI	Multidisciplinary Digital Publishing Institute
DOAJ	Directory of open access journals
TLA	Three letter acronym
LD	linear dichroism
